# Attention for speaking: domain-general control from the anterior cingulate cortex in spoken word production

**DOI:** 10.3389/fnhum.2013.00832

**Published:** 2013-12-09

**Authors:** Vitória Piai, Ardi Roelofs, Daniel J. Acheson, Atsuko Takashima

**Affiliations:** ^1^Donders Institute for Brain, Cognition and Behaviour, Radboud University NijmegenNijmegen, Netherlands; ^2^International Max Planck Research School for Language SciencesNijmegen, Netherlands; ^3^Neurobiology of Language Department, Max Planck Institute for PsycholinguisticsNijmegen, Netherlands; ^4^Behavioural Science Institute, Radboud University NijmegenNijmegen, Netherlands

**Keywords:** attentional control, anterior cingulate cortex, superior temporal cortex, picture-word interference, Simon, Stroop, word production

## Abstract

Accumulating evidence suggests that some degree of attentional control is required to regulate and monitor processes underlying speaking. Although progress has been made in delineating the neural substrates of the core language processes involved in speaking, substrates associated with regulatory and monitoring processes have remained relatively underspecified. We report the results of an fMRI study examining the neural substrates related to performance in three attention-demanding tasks varying in the amount of linguistic processing: vocal picture naming while ignoring distractors (picture-word interference, PWI); vocal color naming while ignoring distractors (Stroop); and manual object discrimination while ignoring spatial position (Simon task). All three tasks had congruent and incongruent stimuli, while PWI and Stroop also had neutral stimuli. Analyses focusing on common activation across tasks identified a portion of the dorsal anterior cingulate cortex (ACC) that was active in incongruent trials for all three tasks, suggesting that this region subserves a domain-general attentional control function. In the language tasks, this area showed increased activity for incongruent relative to congruent stimuli, consistent with the involvement of domain-general mechanisms of attentional control in word production. The two language tasks also showed activity in anterior-superior temporal gyrus (STG). Activity increased for neutral PWI stimuli (picture and word did not share the same semantic category) relative to incongruent (categorically related) and congruent stimuli. This finding is consistent with the involvement of language-specific areas in word production, possibly related to retrieval of lexical-semantic information from memory. The current results thus suggest that in addition to engaging language-specific areas for core linguistic processes, speaking also engages the ACC, a region that is likely implementing domain-general attentional control.

## Introduction

Accumulating evidence suggests that speakers need to engage attentional control for certain language processes (e.g., Ferreira and Pashler, 2002; Roelofs and Hagoort, 2002; Roelofs, 2003, 2008; Roelofs and Piai, 2011; Piai and Roelofs, 2013). Attentional control refers to the regulatory and monitoring processes that ensure that our actions are in accordance with our goals, especially in the face of distraction (e.g., Posner and Petersen, 1990; Roelofs, 2003). For example, when planning a word or a multi-word utterance, speakers need to prevent interference from concurrent information in the environment, such as speech from an interlocutor or visual input from objects surrounding the referent. The object that one wants to refer to may have more than one name, in which case top-down regulation is needed to resolve the conflict between alternative responses. Attentional control also includes self-monitoring, through which speakers assess whether planning and performance are consistent with intent (e.g., Levelt et al., 1999; Hartsuiker and Kolk, 2001; Roelofs, 2004; Christoffels et al., 2007; van de Ven et al., 2009). For example, Levelt (1989) suggests that “Message construction is controlled processing, and so is monitoring” (p. 21).

The present study was designed to address the extent to which these controlled processes may be language-specific or domain-general. In particular, we used functional magnetic resonance imaging (fMRI) to examine brain activity associated with performance in three tasks varying both in the amount of attentional control and in the amount of linguistic processing needed: vocal picture naming with distractor words (picture-word interference, PWI); vocal color naming with distractor words (Stroop); and object discrimination using manual responding with spatial compatibility (Simon task). All three tasks contained stimuli with two dimensions that were either congruent or conflicting with each other, and required responding to a relevant dimension while ignoring an irrelevant one. Given that such conflict often leads to increases in error rates or to the selection of an inappropriate response, people must constantly monitor and regulate their performance (e.g., Posner and Petersen, 1990; Petersen and Posner, 2012). Thus, these three tasks measure the extent to which attentional control is required to select a target response (e.g., Posner and Petersen, 1990; Roelofs, 2003; Hommel, 2011; Petersen and Posner, 2012), with conflicting stimulus dimensions in the incongruent condition increasing response time (RT) relative to neutral and congruent trials.

Attentional control functions have been extensively studied with the Stroop (1935; see also MacLeod, 1991) and Simon tasks (Simon and Small, 1969; see also Hommel, 2011). In the Stroop task, participants name the ink color of words, with the ink color being either congruent (e.g., *red* printed in red ink), incongruent (e.g., *blue* in red ink), or neutral (e.g., *dream* in red ink) with respect to the written word. In the Simon task, participants are instructed to respond to a color or to the identity of an object with lateralized button presses (e.g., press right for a triangle and left for a square), and spatial congruency is manipulated either by presenting the object in the same (i.e., congruent) or opposite (i.e., incongruent) spatial position relative to the response. To examine attentional control functions in spoken word production, tasks such as Stroop and PWI can be used. In the PWI task (Rosinski, 1977; see for review Glaser, 1992), participants name pictures while trying to ignore superimposed distractor words that are, for example, semantically related (e.g., pictured *car* with distractor *bus*), semantically unrelated (e.g., pictured *car*, distractor *table*), or identical to the picture name (e.g., pictured *car*, distractor *car*). Thus, in addition to providing insight into lexical access, PWI is often seen as an experimental method that allows us to examine monitoring and regulation processes in spoken word production (e.g., Lupker, 1979; Glaser and Düngelhoff, 1984; MacLeod, 1991; Roelofs, 2003; Dhooge and Hartsuiker, 2011). In the remainder of this article, we refer to the semantically related condition as *incongruent*, the unrelated as *neutral*, and the identical condition as *congruent*.

A network of brain areas has commonly been implicated in attentional control functions, as measured with the Stroop and Simon tasks (e.g., Peterson et al., 2002; Fan et al., 2003; Liu et al., 2004). In particular, the effects of conflict in these tasks, i.e., more activity for incongruent relative to congruent stimuli, have been co-localized to the lateral prefrontal cortex (PFC) and the dorsal anterior cingulate cortex (ACC) (Fan et al., 2003; Liu et al., 2004). The dorsal ACC includes Brodmann areas 24 and 32 (Devinsky et al., 1995; Paus, 2001; Ridderinkhof et al., 2004), referred to as “anterior” and “mid” cingulate in the Automated Anatomical Labeling (AAL) template (Tzourio-Mazoyer et al., 2002). The dorsal ACC is part of a frontoparietal network underlying domain-general attentional control (e.g., Duncan, 2010; Barbey et al., 2012; Niendam et al., 2012), both at the task and response level (Aarts et al., 2009). Although the exact function of the dorsal ACC within this network is still debated in the literature (e.g., conflict monitoring, Botvinick et al., 2004; response selection, Awh and Gehring, 1999; top-down regulation of selection processes, Roelofs et al., 2006; Aarts et al., 2008; see also Alexander and Brown, 2011 for a recent proposal encompassing several other accounts), all theoretical frameworks acknowledge that the engagement of the dorsal ACC increases with incongruent relative to congruent or neutral stimuli.

In the past few years, significant progress has been made in delineating the neural substrates of the core language processes underlying speaking through the use of tasks such as picture naming, word generation, and word/pseudoword reading (for overviews see Indefrey and Levelt, 2004; Indefrey, 2011; Price, 2012). Despite this progress, the neural substrates associated with the processes of regulating and monitoring language production have remained relatively underspecified (cf. Indefrey, 2011; for recent advances, see Nozari et al., 2011; Riès et al., 2011), in part because the manipulations and comparisons within these tasks may not have been sensitive to attentional control functions. As concerns vocal utterances, the ACC plays an important role in controlling the initiation and suppression of non-verbal vocalizations in humans, such as laughing and crying (Jürgens, 2002). Because of its connections with the lateral PFC, which is involved in broad aspects of top-down control (e.g., Paus, 2001; Petrides, 2005), it has been argued that the ACC has the appropriate characteristics to mediate the attentional control necessary for producing language (e.g., Roelofs, 2008). Evidence for this proposal comes, for example, from a review of two decades of language production neuroimaging research, indicating a critical role for the dorsal ACC during word selection in the context of non-target words (Price, 2012).

Despite this evidence, some important questions about the role of the dorsal ACC in language production have remained unanswered. In their meta-analysis of neuroimaging studies on word production, Indefrey and Levelt (2004) identified the mid-cingulate (part of the dorsal ACC more commonly defined) as one of the brain areas that are active in all production tasks examined (i.e., picture naming, word generation, and word/pseudoword reading). This suggests that the dorsal ACC may implement a production-general function (i.e., regulation and monitoring) rather than making a specific contribution to core language production processes (i.e., conceptual preparation, lexical selection, and word-form encoding). However, whether the production-general contribution of the dorsal ACC is also domain-general (i.e., also engaged outside the language domain) could not be assessed in the meta-analysis of Indefrey and Levelt. Moreover, it is still unclear whether regulation and monitoring processes in word production, as measured by the PWI task, involve the dorsal ACC. The first study to report ACC activity in PWI compared categorically related (incongruent) picture-distractor pairs with a control picture-distractor pair (i.e., a string of Xs) (de Zubicaray et al., 2001). Note that the comparison between categorically related picture-word pairs and pictures paired with a string of Xs concerns a contrast between a word and non-word condition rather than between different word conditions (e.g., semantically related and unrelated words). Subsequent studies examining the contrast between categorically related and unrelated picture-word pairs (often referred to as the *semantic effect*) failed to observe modulations of ACC activity as a function of distractor type (Spalek and Thompson-Schill, 2008; de Zubicaray and McMahon, 2009; de Zubicaray et al., 2013). Importantly, the portion of the ACC that was sensitive to distractor type in the study of de Zubicaray et al. (2001) does not correspond to areas previously associated with domain-general control, but rather to those observed in tasks involving the processing and control over emotion, reward, and pain (see Torta and Cauda, 2011) in the anterior portion of the ACC. Thus, it is unclear whether the system for attentional control in word production, commonly measured with the PWI task, is part of the same domain-general, attentional control system that has been implicated outside of language.

An additional goal of the present study was to determine whether common brain activation associated with lexical-semantic processing in word production can be found for the PWI and Stroop tasks. Although retrieval of words from long-term memory may rely on general processes for retrieving diverse information from memory, the storage of lexical-semantic knowledge has been mainly associated with the left superior and middle temporal cortex (see for overviews Indefrey and Levelt, 2004; Price, 2012). In an extensive lesion-deficit analysis concerning semantic errors in picture naming by individuals with post-stroke aphasia, Schwartz et al. (2009) identified the left anterior temporal cortex as the brain area that is critically involved in mapping concepts onto words in production (i.e., conceptually driven “lemma retrieval”). This anterior temporal area included the mid-temporal region identified by Indefrey and Levelt (2004) as being involved in conceptually driven word retrieval, providing converging evidence for the functional role assigned to this area. PWI studies have consistently revealed sensitivity of the left superior temporal gyrus (STG) and middle temporal gyrus (MTG) activity to experimental manipulations (de Zubicaray et al., 2001, 2002, 2013; de Zubicaray and McMahon, 2009), but in Stroop studies, activity in left temporal cortex is generally absent (e.g., Bench et al., 1993; Banich et al., 2000). Despite these previous results, it seems reasonable to predict that both tasks might activate elements of the temporal cortex as the distracting information is lexical-semantic in nature.

To recapitulate, the present study was designed to elucidate the inconclusive evidence for the involvement of a domain-general control mechanism, possibly supported by the dorsal ACC, in language production. Furthermore, we also investigated language-specific activity in the left superior and middle temporal cortex, areas shown to be consistently involved in lexical-semantic processes in language production (Indefrey and Levelt, 2004; Indefrey, 2011). We used three tasks that are known to require attentional control, but crucially two of them were language tasks with vocal responding (PWI and Stroop), whereas the third was a spatial congruency task requiring manual responding (Simon). By examining the activity in the dorsal ACC that is common to all three tasks, we aimed to identify a domain-general portion of the cingulate cortex that is active with incongruent (i.e., more difficult) trials. If domain-general control is involved in language production, then such a common dorsal ACC area should be found. Furthermore, we also investigated the activity in the left superior and middle temporal cortex, areas shown to be consistently involved in lexical-semantic retrieval in language production (Indefrey and Levelt, 2004; Indefrey, 2011).

## Methods

### Participants

The experiment was approved by the Ethics Committee for Behavioral Research of the Social Sciences Faculty at Radboud University Nijmegen. Twenty-six young adults (mean age = 21.2 years, range = 18–29) from the pool of the Radboud University Nijmegen participated in the experiment for monetary compensation or course credits. All participants gave informed written consent to their participation after the nature and possible consequences of the study were explained. Three female participants were excluded from the analyses for the following reasons. One participant revealed having dyslexia after the data were acquired; for another participant, a technical failure caused an imprecision in the registration of the time parameters; one participant was discarded for excessive movement in the scanner (>6 mm). The remaining 23 participants (11 male) were right-handed, native speakers of Dutch with normal or corrected-to-normal vision, and no history of neurological or reading deficits.

### Materials and design

#### Picture-word interference task

Forty pictures were selected from the picture database of the Max Planck Institute for Psycholinguistics, Nijmegen, together with their basic-level names in Dutch. The pictures belonged to ten different semantic categories with four objects pertaining to each category. All pictures were white line drawings on a black background. The pictures subtended between 1° and 1.3° of the participant's visual angle. A list of the materials can be found in the Appendix. Three picture-word conditions were created. In the incongruent (categorically related) condition, each target picture was combined with a distractor word from the same semantic category (i.e., the distractor words were the names of the other category-coordinate pictured objects from our materials). For the neutral (categorically unrelated) condition, the pictures were re-combined with the names of the pictures from the other semantic categories. Finally, in the congruent condition, the distractor words were the Dutch name of the pictures. Thus, all distractor words belonged to the response set and distractor type was varied within participants and within items. Each picture appeared once in each condition, totalling 40 trials per condition. The distractors were presented in font Arial size 30 in white, centered on the picture. The picture-word trials were randomized using Mix (Van Casteren and Davis, 2006), with one unique list per participant. Participants were instructed to name the picture and to ignore the distractor word.

#### Stroop task

All words were presented in red, green, and blue font. There were three Stroop conditions: congruent, incongruent, and neutral. In the incongruent condition, the color words (*red, green*, and *blue*) were displayed in an incongruent ink color (e.g., *red* was presented in green and in blue, etc.). In the neutral condition, the Dutch words *taak* (“task”), *droom* (“dream”), and *klant* (“client”) appeared 5 times in each ink color. In the congruent condition, each color word appeared in its corresponding ink color. Each color word appeared 15 times in each condition, totalling 45 trials per condition. The Stroop stimuli were presented in the center of the screen in Arial font size 20, subtending between 1° and 1.3° of the participant's visual angle. The color-word trials were randomized using Mix (Van Casteren and Davis, 2006), with one unique list per participant. Participants were instructed to name the ink color of the words.

#### Simon task

A square and a triangle were used as white line drawings presented on a black background, subtending about 3° of the participants' visual angle. Half of the participants were instructed to press a button with their left index finger in response to squares and another button with their right index finger to triangles. The other half of the participants received the opposite shape-button press mapping. Each shape appeared 33 times to the left of a centred fixation cross and 33 times to the right, yielding 66 congruent- and 66 incongruent-location trials. Note that this task lacked a neutral condition as this is not typically employed within this task. All 132 trials were randomized using Mix (Van Casteren and Davis, 2006), with one unique list per participant. For the Simon task, two button boxes were resting on the participant's body, one near each hand.

### Procedure and apparatus

Outside the scanner, participants read the instructions and were familiarized with the pictures and the names to be used in the experiment. Both speed and accuracy were emphasized for all three tasks. Next, participants practiced each task with eight trials (PWI and Stroop) or 14 trials (Simon) in the same order they would perform them in the scanner, i.e., PWI, Stroop, Simon task. For the PWI task, two line drawings (heart and star) were selected as practice items. For the Stroop and Simon tasks, the same items were used for the practice and experimental sessions.

The presentation of stimuli (screen resolution 1024 × 768 × 32, 60 Hz refresh rate) and the recording of responses were controlled by Presentation Software 14.1 (Neurobehavioral Systems, Albany, CA). A noise-cancelling microphone, placed above the participant's mouth, was connected to the presentation computer, enabling the recording of vocal responses and the measurement of vocal response latencies. The experiment started with the PWI task. A prompt on the screen indicated the end of one task and the beginning of the next task, with the instructions presented once more for 20 s. The Stroop task followed the PWI task, and the Simon task was performed last. For all three tasks, a trial started with the presentation of a fixation cross in the center of the screen for 500 ms. Next, the stimulus was displayed for 1 s. For PWI and Stroop stimuli, they were displayed in the center of the screen. For the Simon task, the stimuli were presented either to the right or to the left of the fixation cross, depending on the Simon condition of the trial. A black screen followed for the duration of the jitter period (varying between 2.4 and 6 s, following a normal distribution, randomly assigned to each trial). The registration of the vocal and manual responses started as soon as the stimuli were displayed and lasted until the next trial started. For each task, the stimuli were presented in three blocks with breaks of 20 s between blocks.

### Data acquisition

Participants were scanned with a 1.5-T Siemens Avanto Scanner with a 32-channel head coil. For the acquisition of the functional data, we used a parallel-acquired inhomogeneity-desensitized fMRI sequence (Poser et al., 2006), which is a multiecho echo-planar imaging sequence that reduces image artefacts and is therefore suitable for acquiring data of participants while they speak (e.g., Menenti et al., 2011; Segaert et al., 2012). In this sequence, the images are acquired at multiple time echoes (TEs) following a single excitation. The time repetition (TR) used was 2.31 s, with the five TEs acquired at 8.3, 27.6, 37, 46, and 55 ms (echo spacing = 0.5 ms, flip angle = 80°). Each volume comprised 36 slices of 3 mm thickness [ascending slice acquisition, voxel size = 3.5 × 3.5 × 3 mm^3^, slice gap = 17%, field of view (FOV) = 224 mm, matrix = 64 × 64]. GRAPPA parallel imaging was used (acceleration factor = 3). Functional scans were acquired in one run. First, 30 volumes were acquired and used for weight calculation of each of the echoes (pre-task volumes), followed by the three tasks one after the other.

For the anatomical MRI, T1-weighted images were acquired using a magnetization-prepared, rapid-acquisition gradient echo sequence (MPRAGE; TR = 2.25 s, TE = 2.95 ms, echo spacing = 8.7 ms, flip angle = 15°). We acquired 176 sagittal slices (isotropic voxel size = 1 mm^3^, FOV = 256 mm, matrix = 256 × 256).

### Behavioral data analysis

For each trial of the PWI and Stroop tasks, the experimenter evaluated the participants' vocal responses. Trials that contained a disfluent response, a wrong pronunciation of the word, or a wrong response word were coded as errors and subsequently excluded from the statistical analyses of the naming RTs. Errors in the Simon task were also excluded from the statistical analysis of the manual RTs. Vocal RTs shorter than 200 ms and manual RTs shorter than 100 ms were also excluded from the analyses.

RTs were averaged over trials per condition and per participant and submitted to by-participant analyses of variance (ANOVA) for the Simon and Stroop tasks separately, and additionally to by-item ANOVA for the PWI task, with stimulus type (neutral, incongruent, congruent) as the independent variable. Planned contrasts were examined with paired *t*-tests (two-tailed). Errors were submitted to logistic regression analyses on single-trial data. For the relevant contrasts (i.e., incongruent vs. congruent, incongruent vs. neutral), 95% confidence intervals (CI) around the mean difference are reported, as well as Cohen's *d* (a measure of effect size), calculated as the difference between two conditions divided by the square root of the averaged variance of the three conditions (Cumming, 2012). Due to technical failures, vocal RTs were not registered for six participants and manual RTs were not registered for one participant (errors were registered). Thus, the statistical analyses of the vocal responses comprised 17 participants and the analyses of the manual responses comprised 22 participants.

### fMRI data pre-processing

The pre-processing steps were conducted using Matlab and SPM8 (www.fil.ion.ucl.ac.uk/spm/software/spm8). First, all volumes were realigned to the first volume and re-sliced. Then the five echoes of each volume were combined to yield one volume per TR using an in-house Matlab script (see for details Poser et al., 2006). For each voxel, optimal weighting for the five echoes were calculated from the 30 pre-task volumes, and the weighting values were applied to the rest of the functional volumes resulting in one volume per TR. Then these images were slice-time corrected to the first slice. The participant's mean image of the functional run after realignment was co-registered with the participant's anatomical volume. Finally, the functional and anatomical images were spatially normalized to Montreal Neurological Institute (MNI) space and smoothed (3D isotropic Gaussian smoothing kernel, full-width at half-maximum = 8 mm).

### fMRI data analysis

Statistical analyses were performed within a general linear model (GLM) framework. For the analysis on individual participants' data, the model included eight regressors timelocked to the onset of each condition of each task (PWI incongruent, PWI neutral, PWI congruent, Stroop incongruent, Stroop neutral, Stroop congruent, Simon incongruent, and Simon congruent), one regressor for trials in which an error was made, and one regressor to model the intra- and inter-task period. The onsets of each event were modeled as a gamma response, or stick-function (i.e., duration = 0) temporally convolved with the canonical hemodynamic response function along with the first temporal derivative. The model also included the six motion parameters and their first derivatives to account for residual movement-related artefacts. Since participants were overtly producing the words during the PWI and Stroop tasks, we specifically included the first derivatives of the motion parameters to account for signals that might be affected by sudden movements due to overt responses. A high pass filter was implemented (1/128 Hz cutoff) to account for slow drifts of the signal. The effects were estimated with a subject-specific fixed-effects model. (We also modeled the RT as durations for each of the trials, but given that the results were quite similar to the ones reported below and we did not have the RTs for all participants, these results are not reported here).

Specific contrasts of interest were calculated for each participant and these contrast images were used as random variables on the group level. All clusters reported as significant had voxels thresholded at *p* = 0.001 (uncorrected), with the cluster-size statistics thresholded at *p* ≤ 0.05 (family-wise error corrected) (Hayasaka and Nichols, 2003). First, we looked into areas that were significant in a whole-brain analysis. Since we were interested in domain-general activations, we localized shared areas that were active in all three tasks. For this aim, ANOVAs were performed on participants' individual contrast images with task and stimulus type as independent variables. We then conducted a “conjunction analysis” by identifying overlapping voxels that were above the threshold (voxel level *p* = 0.001, uncorrected) in each of the incongruent condition of all three tasks. For the linguistic-vocal tasks, images of each stimulus type were contrasted for each task separately using paired *t*-tests on the group level.

#### ROI analyses

Given our interest in the involvement of the dorsal ACC, STG and MTG, a region of interest (ROI) analysis was performed by restricting our search volume within these ROIs defined anatomically using the AAL template (Tzourio-Mazoyer et al., 2002). Furthermore, we were interested in the specific part of the dorsal ACC that was active during the conflict trials in all three tasks. For this, a conjunction analysis was performed within the bilateral cingulate cortices in the same way as reported above. The dorsal portion of the cingulate cortex that was commonly active in all three incongruent conditions, as shown in this conjunction analysis, was selected as the functional Cingulate ROI. To determine the involvement of this specific Cingulate ROI in the tasks separately, the beta weights from the functional Cingulate ROI were extracted and averaged for each participant and condition separately using the MarsBar toolbox (Brett et al., 2002). Paired *t*-tests were used to test the conflict conditions in a pair-wise fashion for each task separately. Since we had an a priori hypothesis that the congruent conditions would elicit the least conflict, one-tailed tests were used.

For the linguistic-vocal tasks, the ROI analyses comprised the left superior and middle temporal cortex (Indefrey and Levelt, 2004), according to the AAL template. The Stroop task showed a significant effect for incongruent > congruent condition in the left temporal cortex. To observe activity differences between conditions for the PWI task in this area, we extracted averaged beta values of each PWI condition from this functional ROI for each participant using MarsBar. Paired *t*-tests (two tailed) were then used to test the conditions in a pair-wise fashion for the PWI task.

## Results

### Behavioral data

Table 1 presents the mean RTs and standard deviations for correct responses and the error rates as a function of stimulus type and task.

**Table 1:** **Mean response time (M) and standard deviation (*SD*) in milliseconds, and percent error (E%) as a function of stimulus type in each task**.

	**PWI**			**Stroop**			**Simon**		
**Stimulus type**	**M**	***SD***	**E%**	**M**	***SD***	**E%**	**M**	***SD***	**E%**
Incongruent	971	171	5.3	852	152	2.9	508	146	5.9
Congruent	853	145	2.9	759	127	0.7	464	145	3.2
Neutral	946	163	4.9	794	129	0.6			

Mean and standard deviation calculated over participants' single-trial data. PWI, picture-word interference.

#### Errors

Table 2 presents the results of the logistic regression analysis on the errors. In sum, in the PWI task, errors were more likely in the incongruent than in the congruent condition but equally likely in the neutral condition, and more likely in the neutral than in the congruent condition. In the Stroop task, errors were more likely in the incongruent than in the congruent and in the neutral conditions, but equally likely in the neutral and congruent conditions. Finally, in the Simon task, errors were more likely in the incongruent than in the congruent condition.

**Table 2:** **Results of the logistic regression analysis on the errors for the three tasks**.

**Contrast**	**log-odds**	**ß *coeff***	***S.E.***	***Wald Z***	***p***
**PWI**					
inc—con	1.9	0.7	0.3	2.5	0.012
inc—neu	−	0.1	0.2	0.4	0.694
neu—con	1.8	0.6	0.3	2.2	0.031
**STROOP**					
inc—con	4.1	1.4	0.4	3.3	0.001
inc—neu	4.7	1.6	0.5	3.4	0.001
neu—con	–	0.2	0.5	0.3	0.781
**SIMON**					
inc—con	1.9	0.6	0.2	3.4	0.001

A dash indicates equal log-odds. coeff, coefficient; con, congruent; inc, incongruent; neu, neutral; PWI, picture-word interference.

#### RTs

Table 3 presents the results of the main effects of stimulus type, which was statistically significant for all three tasks. Table 4 presents the results of the pair-wise comparisons of condition for the three tasks. In sum, for all three tasks, RTs in the incongruent condition were longer than in the congruent and neutral (PWI and Stroop) conditions. Vocal RTs were also longer in the neutral than in the congruent condition.

**Table 3:** **Results of the analyses of variance on response times for the main effect of stimulus type in the picture-word interference, Stroop, and Simon tasks**.

**Main effect stimulus type**	***F***	**df**	***p***
PWI	41.4/103.2	2, 32/2, 78	<0.001
Stroop	50.6	2, 32	<0.001
Simon	72.3	1, 21	<0.001

For the picture-word interference (PWI) task, by-participant and by-item values are shown side-by-side, separated by the slash.

**Table 4:** **Results of the pair-wise comparisons of response times between conditions for the picture-word interference, Stroop, and Simon tasks**.

**contrast**	**diff**	***t* (df)**	***p***	**95% CI**	***d***
**PWI**					
inc—con	118	7.4 (16)/13.7 (39)	<0.001/<0.001	[87, 158]	0.74
inc—neu	25	3.2 (16)/2.4 (39)	0.005/0.019	[10, 47]	0.16
neu—con	93	6.1 (16)/12.5 (39)	<0.001/<0.001	[61, 127]	0.58
**STROOP**					
inc—con	93	8.1 (16)	<0.001	[72, 124]	0.68
inc—neu	58	6.2 (16)	<0.001	[42, 84]	0.43
neu—con	35	5.3 (16)	<0.001	[21, 49]	0.25
**SIMON**					
inc—con	44	8.5 (21)	<0.001	[34, 56]	0.31

For the picture-word interference (PWI) task, by-participant and by-item values are shown side-by-side, separated by the slash. CI, confidence interval; diff, difference in milliseconds; inc, incongruent; con, congruent; neu, neutral; PWI, picture-word interference.

### fMRI data

#### Cross-domain activity

**Figure 1:**
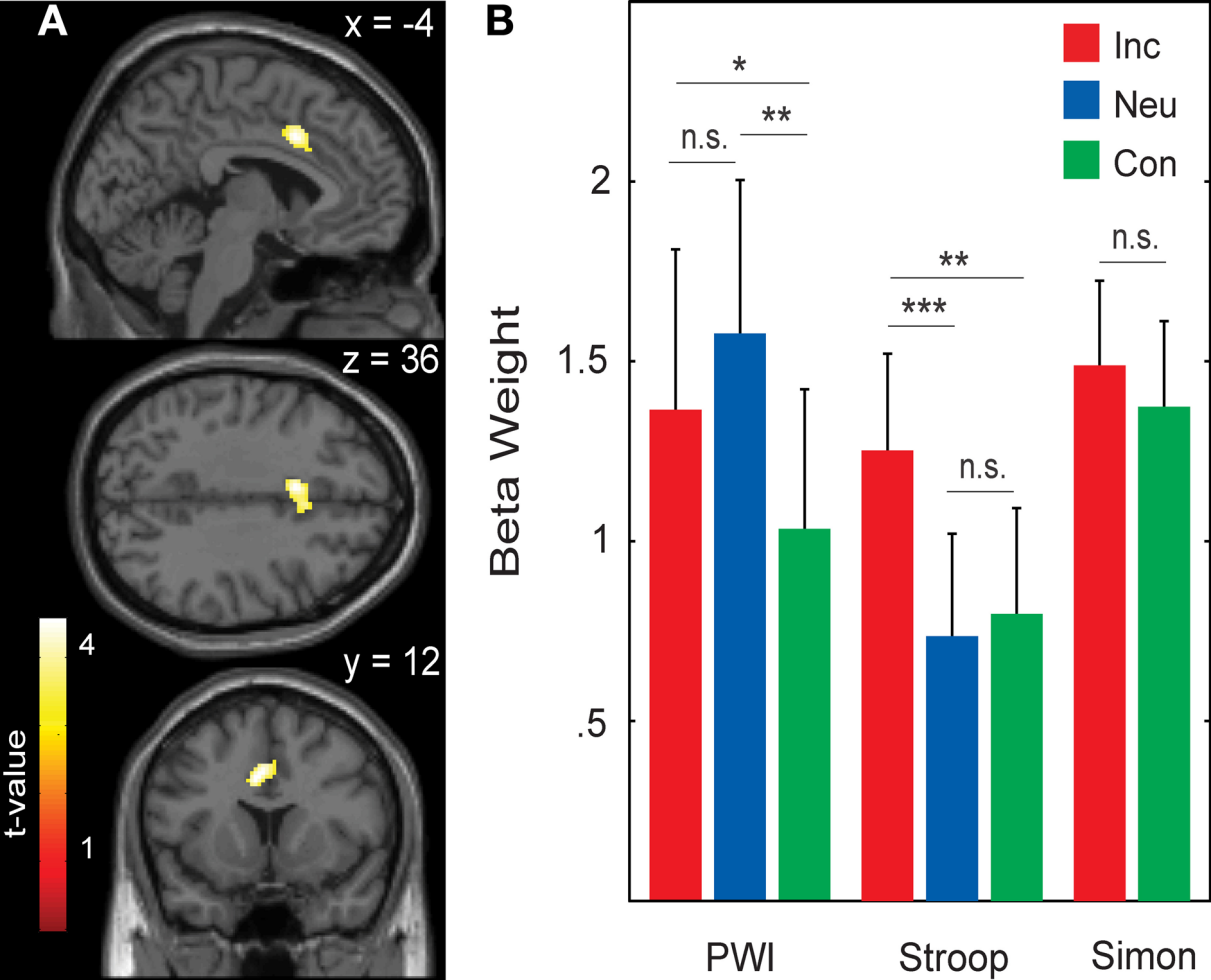
**(A)** Activity common to incongruent stimuli in the picture-word interference (PWI), Stroop, and Simon tasks in the anterior cingulate cortex (BA 24; peak MNI: −4, 12, 36; and BA 32; peak MNI: 4, 18, 36). **(B)** Averaged beta weights of active voxels in the anterior cingulate cortex (shown in **A**) as a function of task and stimulus type. Inc, incongruent; Neu, neutral; Con, congruent; n.s., non-significant. Error bars represent the standard error of the mean. ^*^p-values ≤ 0.05, ^**^p-values ≤ 0.01, ^***^p-values ≤ 0.005.

**Figure 2:**
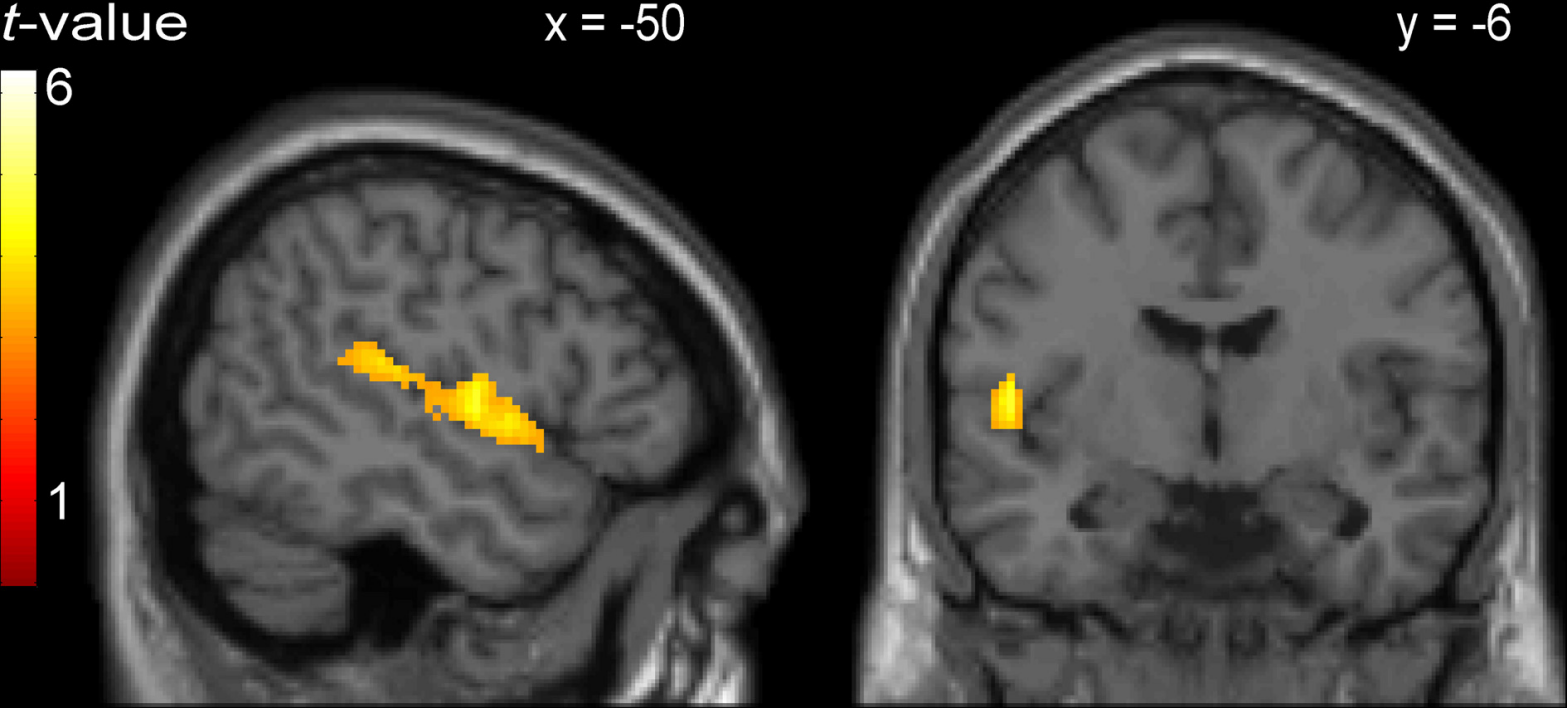
**Activity common to incongruent stimuli in the picture-word interference, Stroop, and Simon tasks in a cluster comprising left Rolandic operculum (BA 22; peak MNI: −50, −6, 4) and left superior temporal gyrus**.

Areas that were commonly activated by incongruent stimuli in all three tasks in the whole-brain analysis are shown in Table 5, Figures 1A, 2. The incongruent stimuli in all three tasks commonly activated the cerebellum (bilaterally), a large cluster in the left Rolandic operculum and STG (Figure 2), and the dorsal ACC (Figure 1A). Furthermore, in line with the whole brain analysis, two peaks of activity were observed in the dorsal ACC (BA 24; MNI: −4, 12, 36; and BA 32; MNI: 4, 18, 36) in the Cingulate ROI analysis, shown in the lower part of Table 5.

**Table 5:** **Statistically significant activations in the whole-brain and ROI analyses for the conjunction of the PWI, Stroop, and Simon tasks**.

**Cluster**		**Voxel**			**MNIspace *x*,* y*,* z* (mm)**	**Anatomical region (AAL)**
***p*(cor)**	**Size**	***t* value**	***z* value**	***p*(unc)**		
**WHOLE-BRAIN ANALYSIS**						
0.000	2720	6.24	5.83	<0.001^*^	30, −54, 28	r cerebellum
		6.00	5.64	<0.001^*^	−28, −56, −26	l cerebellum
		5.08	4.84	<0.001^*^	−18, −56, −22	l cerebellum
0.001	625	4.51	4.34	<0.001	−50, −6, 4	l Rolandic operculum
		4.11	3.98	<0.001	−46, −30, 16	l superior temporal g.
		4.03	3.90	<0.001	−48, 4, 0	l superior temporal g.
0.041	260	4.55	4.37	<0.001	−4, 12, 36	mid cingulate gyrus
		4.13	4.00	<0.001	0, 12, 46	supplem. motor area
		3.96	3.84	<0.001	−2, 4, 50	medial frontal gyrus
**ANATOMICAL ROI ANALYSIS**						
0.009	187	4.55	4.37	<0.001^*^	−4, 12, 36	mid cingulate
		3.89	3.78	<0.001^*^	4, 18, 36	mid cingulate

Voxels thresholded at p = 0.001. For each cluster, coordinates are given for the maximally activated voxel and up to two local maxima more than 8 mm apart. Cluster size corresponds to the number of voxels (2 × 2 × 2 mm) comprising the cluster. The mid cingulate in the AAL template is part of the dorsal ACC as usually defined (Devinsky et al., 1995; Paus, 2001; Ridderinkhof et al., 2004). Cor, Family-wise error (FWE) corrected on the cluster-level; g., gyrus; l, left; r, right; supplem., supplementary; unc, uncorrected.

* Voxel p < 0.05 also when FWE-corrected on the voxel level.

Note that ideally, analyses would have targeted regions showing increased BOLD responses for the incongruent relative to the congruent conditions across all three tasks. However, this analysis proved to be untenable in the present investigation as the BOLD responses in the dorsal ACC in the Simon task were elevated in both congruent and incongruent conditions (see below), preventing us from detecting regions showing increased activity for the incongruent relative to the congruent condition in this task. Thus, we were not able to detect brain areas that were commonly modulated by stimulus type (i.e., incongruent > congruent) across all three tasks. Importantly, the cross-task conjunction of incongruent conditions still entails a contrast, i.e., vs. a low-level baseline. Hence, with this contrast, we detect the activity from the most difficult condition in all three tasks relative to this low-level baseline. This is comparable to the approach taken by Indefrey and Levelt (2004) in their meta-analysis, where activity common to different production tasks was detected by means of a comparison to a low-level baseline.

Figure 1B shows the mean beta weights extracted for each stimulus type in the three tasks from the Cingulate ROI, which was generated from the conjunction of the incongruent conditions across all three tasks. In the Stroop task, dorsal ACC activity was higher with incongruent than with congruent stimuli, *t*_(22)_ = 2.61, *p* = 0.008; and higher with incongruent than neutral stimuli, *t*_(22)_ = 3.02, *p* = 0.003; but similar for neutral and congruent stimuli, *t*_(22)_ < 1. In the PWI task, dorsal ACC activity was higher with incongruent than with congruent stimuli, *t*_(22)_ = 1.99, *p* = 0.030; and higher with neutral than congruent stimuli, *t*_(22)_ = 2.87, *p* = 0.009; but similar for neutral and incongruent stimuli, *t*_(22)_ = 1.43, *p* = 0.083. In the Simon task, elevated dorsal ACC activity did not differ between the incongruent and congruent conditions, *t*_(22)_ < 1. The same pattern of activity was observed in the beta weights when we constrained the analyses to the 17 participants for whom RT data was available.

#### Language-specific activity

**Figure 3:**
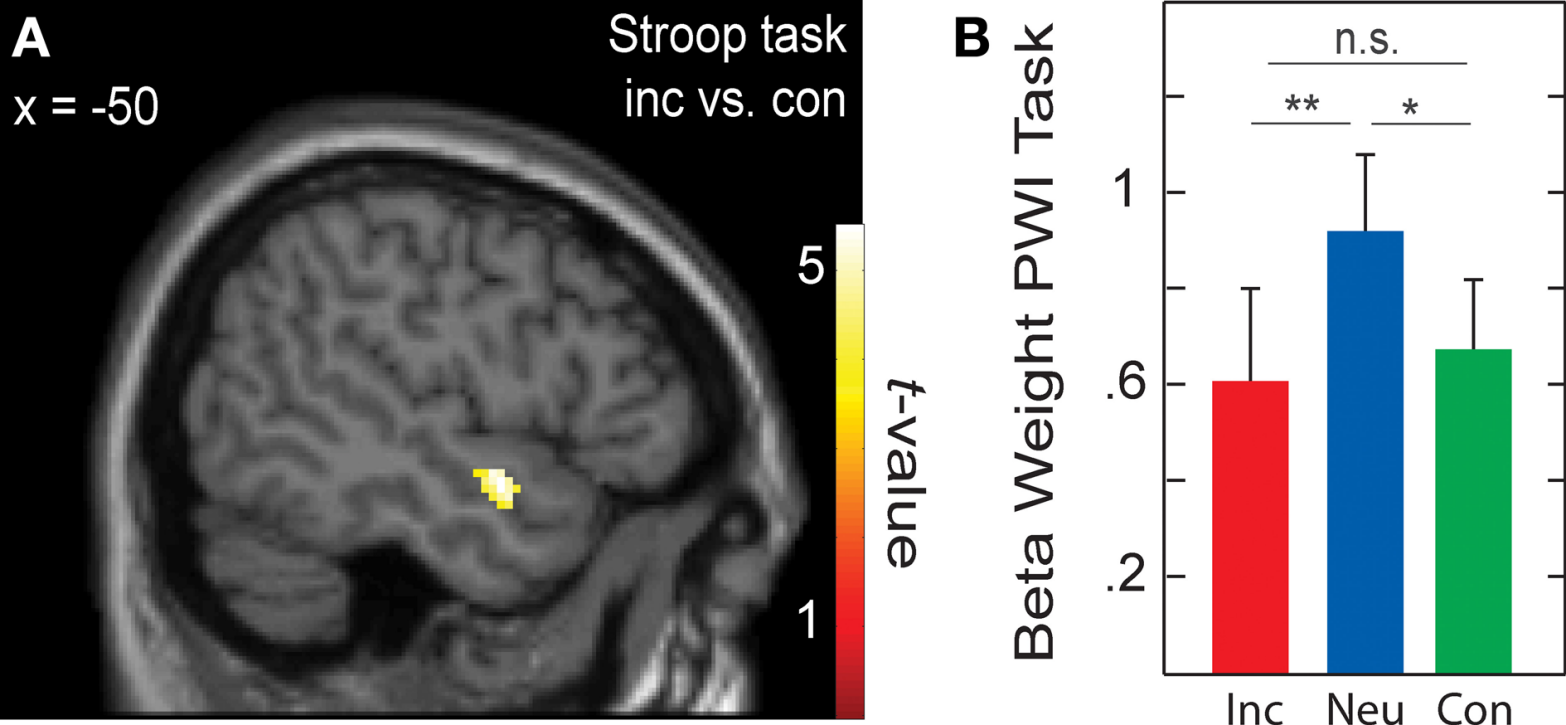
**(A)** Active voxels for incongruent versus congruent in the Stroop task (BA 38; peak MNI: −50, 0, −12; and −46, −10, −12). **(B)** Averaged beta weights of active voxels in **(A)** in the picture-word interference (PWI) task as a function stimulus type. Inc, incongruent; Neu, neutral; Con, congruent; n.s., non-significant. Error bars represent the standard error of the mean. ^*^p-values ≤ 0.05, ^**^p-values ≤ 0.01.

When testing for differences in brain activation between conditions for each task separately with paired *t*-tests, only the Stroop task yielded significant results for the contrasts incongruent > congruent and incongruent > neutral. These results are presented in Table 6 and in Figure 3A. In the whole-brain analysis, shown in the upper part of Table 6, both conflict contrasts (i.e., incongruent vs. neutral and incongruent vs. congruent) showed increased activity in the right inferior frontal gyrus (rIFG). In the Cingulate ROI analysis, shown in the lower part of Table 6, dorsal ACC activations were also increased for incongruent stimuli relative to neutral and congruent stimuli. Interestingly, in the Left Temporal ROI analysis, shown in Figure 3A, activity in the left STG was also increased for incongruent relative to congruent stimuli in the Stroop task. Note that this left STG ROI area (MNI −50, 0, −12 and −46, −10, −12) is slightly more ventral than the left STG area (MNI −46, −30, 16 and −48, 4, 0) that was identified by the conjunction of the incongruent conditions in all three tasks (section Cross-domain activity). That is, this region of the left STG is not activated by the Simon task, suggesting language-specific activation.

**Table 6:** **Statistically significant activations for the Stroop task in the whole-brain and ROI analyses (cingulate and left superior/middle temporal cortex)**.

**Cluster**		**Voxel**			**MNI space x,y,z (mm)**	**Anatomical region (AAL)**
***p*(cor)**	**Size**	***t* value**	***z* value**	***p*(unc)**		
**WHOLE-BRAIN ANALYSIS**						
**Incongruent versus congruent**						
0.000	528	5.70	4.42	<0.001	36, 22, −14	r IFG pars orbitalis
		4.24	3.58	<0.001	38, 28, 2	r IFG pars triangularis
0.025	211	5.57	4.36	<0.001	12, 10, 8	r caudate
		4.03	3.45	<0.001	14, 12, −6	r putamen
		3.84	3.33	<0.001	16, −6, 10	
**Incongruent versus neutral**						
0.007	294	5.41	4.27	<0.001	32, 16, −14	r insula
		4.24	3.59	<0.001	44, 20, 6	r IFG pars triangularis
		4.01	3.44	<0.001	34, 26, 0	r insula
0.001	461	5.37	4.25	<0.001	4, 28, 30	mid cingulate
		4.84	3.95	<0.001	8, 18, 46	r supplem. motor area
		4.79	3.92	<0.001	12, 32, 26	anterior cingulate
**ANATOMICAL ROI ANALYSIS**						
**Incongruent versus congruent**						
0.025	88	4.18	3.55	<0.001	0, 30, 26	anterior cingulate
		3.90	3.36	<0.001	−4, 30, 22	anterior cingulate
		3.86	3.33	<0.001	4, 24, 30	mid cingulate
0.042	65	5.48	4.31	<0.001^*^	−50, 0, −12	l sup. temporal g.
		3.59	3.15	0.001	−46, −10, −12	l superior temporal
**Incongruent versus neutral**						
0.000	370	5.37	4.25	<0.001^*^	4, 28, 30	mid cingulate
		5.11	4.11	<0.001^*^	6, 34, 28	anterior cingulate
		4.79	3.92	<0.001^*^	12, 32, 26	anterior cingulate

Voxels thresholded at p = 0.001. For each cluster, coordinates are given for the maximally activated voxel and up to two local maxima more than 8 mm apart. Cluster size corresponds to the number of voxels (2 × 2 × 2 mm) comprising the cluster. The mid cingulate in the AAL template is part of the dorsal ACC as usually defined (Devinsky et al., 1995; Paus, 2001; Ridderinkhof et al., 2004). Cor, Family-wise error (FWE) corrected on the cluster-level; g., gyrus; IFG, inferior frontal gyrus; l, left; r, right; sup., superior; supplem., supplementary; unc, uncorrected.

* Voxel p < 0.05 also when FWE-corrected on the voxel-level.

To examine language-specific activity in the PWI task, the averaged beta weights within this left STG cluster were extracted, which is shown in Figure 3B. Activity in left STG was higher with neutral than with congruent (identical) stimuli, *t*_(22)_ = 2.31, *p* = 0.030; and higher with neutral than incongruent (categorically related) stimuli, *t*_(22)_ = 2.87, *p* = 0.009; but similar for congruent and incongruent stimuli, *t*_(22)_ < 1. Importantly, activity in this left STG cluster was not significantly increased from baseline for the Simon task (incongruent: beta weight = 0.008, *t*_(22)_ < 1; congruent: beta weight = 0.37, *t*_(22)_ = 1.73, *p* = 0.097), nor did it differ between incongruent and congruent conditions, *t*_(22)_ < 1. The same pattern of activity was observed in the beta weights when we constrained the analyses to the 17 participants for whom RT data was available.

## Discussion

In the present study, we compared three control-demanding tasks, two of which had linguistic stimuli requiring vocal responding (Stroop and PWI), and the third had visual-spatial stimuli requiring manual responding (Simon task). Participants responded to congruent and incongruent stimuli in all three tasks, and in the Stroop and PWI tasks to neutral stimuli as well. Behaviorally, RTs were longer for incongruent than for congruent stimuli in all three tasks. Furthermore, in the linguistic-vocal tasks, RTs were longer for neutral than for congruent stimuli. These results are in line with previous literature for all three tasks (for reviews: PWI: Glaser, 1992; Stroop: MacLeod, 1991; Simon: Hommel, 2011).

Regarding the neuroimaging data, an analysis was performed to identify areas showing increased BOLD responses common to the incongruent condition in all three tasks (cross-domain activation). The areas identified by this conjunction analysis were the bilateral cerebellum, the left Rolandic operculum extending to the left STG, and the dorsal ACC.

Top-down control of task performance has been associated with a frontoparietal network of brain areas, including the lateral prefrontal cortex, the anterior insula/frontal operculum, the pre-supplementary motor area (SMA) and the ACC, and regions in and around the intraparietal sulcus (e.g., Dosenbach et al., 2006; Duncan, 2010; Power et al., 2011; Barbey et al., 2012; Niendam et al., 2012; Petersen and Posner, 2012). Our finding of common activation in the left operculum, SMA, and ACC across incongruent conditions in all tasks is in line with the evidence that a domain-general attentional control system is implemented by frontoparietal areas. Given our specific interest in the involvement of the ACC in speech production, we further examined activity in this area for the language tasks.

### Cross-domain anterior cingulate cortex activity in language tasks

An extensive meta-analysis of the cingulate cortex has linked different portions of this area to different behavioral domains, i.e., attention, action, emotion, language, memory, and pain (Torta and Cauda, 2011). In this meta-analysis, two adjacent regions were shown to be involved in all six domains examined, suggesting the exercise of a general function that is commonly called upon by performance in multiple tasks. Notably, the portion of the cingulate cortex where we observed the common activity across our tasks is a part of this multi-domain area identified by the meta-analysis. The activity we observed in the domain-general portion of the cingulate cortex was common to the incongruent condition of all three tasks, thus, independent of the response modality and nature of the stimuli (linguistic vs. non-linguistic). Therefore, the most plausible account for our results is that this activity reflects a domain-general attentional control function, a proposal that is also in line with the functional interpretation of the frontoparietal network of brain areas (e.g., Dosenbach et al., 2006; Duncan, 2010; Barbey et al., 2012; Niendam et al., 2012; Petersen and Posner, 2012). As indicated previously (section Introduction), researchers have found no agreement about what exactly this domain-general function of the ACC is (e.g., conflict monitoring, top-down regulation) but at least our result shows that the activity in this region is present when controlled responses are required in both linguistic and non-linguistic domains.

The evidence for the involvement of the dorsal ACC in spoken word production has thus far remained inconclusive in the literature. To address this issue, we examined the portion of the dorsal ACC that was activated across tasks for modulations in activity as a function of stimulus type in the language tasks (Stroop and PWI). In the Stroop task, activity was higher for incongruent than for neutral and congruent color words. In the PWI task, activity was higher for incongruent and neutral picture-word pairs relative to congruent pairs. These results provide the first direct neuroimaging evidence for the involvement of a domain-general portion of the cingulate cortex in the control over spoken word production (for a comparison between Stroop and Simon tasks with manual responding see Peterson et al., 2002; Liu et al., 2004). Our results agree with the proposal of Roelofs and colleagues (e.g., Roelofs and Hagoort, 2002; Roelofs, 2003; Roelofs et al., 2006), who argued for a regulation function of the ACC, in line with the evidence for a regulatory role of the ACC in non-verbal vocalizations (Aitken, 1981; Ploog, 1981; Jürgens, 2002, 2009). Moreover, our results also agree with the recent proposal of Nozari et al. (2011), who suggested that the ACC is implicated in self-monitoring in language production, in line with the ACC conflict-detection view (Botvinick et al., 2004). The present results do not allow us to adjudicate between the regulation and monitoring views, so future studies explicitly addressing this issue are needed.

#### Interference effects in behavior and brain activity

We observed a discrepancy in the language tasks between the condition differences in the RTs (incongruent > neutral > congruent) and the beta estimates in the dorsal ACC (see Figure 1). For the Stroop task, the incongruent condition led to an increased BOLD response relative to both the neutral and congruent conditions (incongruent > neutral = congruent), whereas for the PWI task, the incongruent and neutral conditions both had higher BOLD responses than the congruent condition (incongruent = neutral > congruent). Conflict, and thus the amount of conflict detected (Botvinick et al., 2004) or the amount of top-down regulation needed (Roelofs et al., 2006), is thought to be highest in the incongruent condition, followed by the neutral, and then the congruent condition. This pattern was clearly present in the RT data, but not in the neuroimaging data, even when the analyses of the neuroimaging data were constrained to the subjects for whom behavioral data was available. Based on this pattern, it could be argued that the present results do not agree with either the conflict monitoring or the top-down regulation views of ACC function.

The apparent discrepancy between RTs and ACC activity, however, can be resolved (and the theoretical views can be saved) if the magnitude of the conflict effects as evident in the RTs is taken into account. The largest RT effects in the PWI and Stroop tasks (>58 ms on average) are also the effects being detected in the BOLD estimates for each task, whereas the contrasts from the smaller behavioral effects, i.e., on average 25 ms for incongruent vs. neutral in PWI and 35 ms for neutral vs. congruent in Stroop, resulted in no statistically significant differences in the BOLD response. The relatively small behavioral effect sizes may suggest that the discrepancy between the behavioral interference effects and the activity in dorsal ACC may well be a matter of low statistical power. Despite the lack of an exact parallel between condition differences in RTs and dorsal ACC activity, the present results support our claim that a domain-general attentional control mechanism in the dorsal ACC is engaged during spoken word production.

#### Anterior cingulate cortex activity in picture-word interference studies

As mentioned in the introduction, only one PWI study had observed increased dorsal ACC activity for categorically related picture-word stimuli (equivalent to our incongruent condition) relative to a low-level control condition (de Zubicaray et al., 2001), whereas subsequent PWI studies did not observe differential activity in this area for categorically related (incongruent) and unrelated (neutral) picture-word pairs (Spalek and Thompson-Schill, 2008; de Zubicaray and McMahon, 2009; de Zubicaray et al., 2013). Similar to some of these previous results, we also did not observe activation differences in the dorsal ACC for categorically related relative to unrelated picture-word pairs. As discussed above, the difference in the amount of conflict between these two conditions may not have been large enough to give rise to detectable differences in brain activity. However, different from all previous studies, our design also included congruent picture-word pairs, for which conflict is absent. Relative to the congruent condition, conflicting picture-word pairs were associated with increased dorsal ACC activity, in line with the hypothesis that the ACC is involved in attentional control over word production (e.g., conflict monitoring or top-down regulation). Previous fMRI investigations comparing categorically related picture-word pairs with no-conflict pairs (i.e., pictures paired with a string of Xs) observed activity in an orbito-frontal ACC area not previously associated with domain-general control (cf. de Zubicaray et al., 2001; Torta and Cauda, 2011). Thus, our study provides evidence for the involvement of the dorsal ACC in control over word production.

### Language-specific activity

#### Stroop task

The Stroop task has been well studied with fMRI, although the large majority of these studies have used manual responding (e.g., Bench et al., 1993; Banich et al., 2000; Liu et al., 2004; see for a brief overview MacLeod and MacDonald, 2000), rather than vocal responding (e.g., Carter et al., 1995; Brown et al., 1999; Barch et al., 2001). In our task, participants responded overtly to incongruent, neutral, and congruent stimuli. In line with previous literature using manual and vocal responding, an increased BOLD response in the dorsal ACC was observed for incongruent relative to congruent and neutral color words, (e.g., Banich et al., 2000; Barch et al., 2001; Fan et al., 2003; for an overview see also MacLeod and MacDonald, 2000). Moreover, the dorsal ACC coordinates we obtained are similar to those obtained by de Zubicaray et al. (2002) when contrasting phonologically related with unrelated picture-word pairs in PWI. Regarding other areas, rIFG and insular activity was also increased for incongruent relative to neutral and congruent stimuli, which is also consistent with previous studies using manual responding (e.g., Peterson et al., 2002; Floden et al., 2011). Earlier studies have suggested that the rIFG is involved in inhibition (e.g., Aron et al., 2004) or the detection of salient or task relevant cues indicating the need for top-down regulation (e.g., Hampshire et al., 2007). Our findings are compatible with both views. However, the literature suggests that the inhibition function implemented by rIFG is domain-general, whereas we observed activity in this area only related to the language tasks. This finding is consistent with the view that inhibition is not necessarily engaged to resolve conflict and can be optionally employed (Verhoef et al., 2009; Roelofs et al., 2011).

In addition to the areas that were common to the Stroop contrasts (incongruent vs. congruent and incongruent vs. neutral), increased BOLD responses were also observed in the right striatum (caudate and putamen) for incongruent relative to congruent stimuli. This finding is in line with the evidence that the caudate nucleus and the putamen are among the primary subcortical areas that underlie attentional control (e.g., Aarts et al., 2010; Wiecki and Frank, 2013), both at the task and response levels (Aarts et al., 2009). These results thus suggest that speech production, like other motor tasks, engages a frontal-striatal network implicated in attentional control. Finally, we also observed increased BOLD responses in the left anterior STG for incongruent relative to congruent stimuli, a less common finding in the literature (e.g., Fan et al., 2003). We will elaborate on this left STG activation in the next section.

#### Picture-word interference task and left temporal cortex

For the left anterior STG area showing BOLD response differences in the Stroop task, activity was increased for neutral (categorically unrelated) relative to the incongruent (categorically related) and congruent stimuli in the PWI task. The STG area we observed is located within the left anterior temporal lobe, a structure crucial for semantic memory (Patterson et al., 2007; Binder et al., 2009; Visser et al., 2010; Bonner and Price, 2013), including the mapping of concepts onto words in production (Indefrey and Levelt, 2004; Schwartz et al., 2009). Furthermore, our left temporal cortex activity is similar to a previous report of a PWI study also using categorically related and unrelated picture-word pairs (de Zubicaray et al., 2013). In that study, the left MTG activity was also interpreted in terms of lexical-semantic memory (Indefrey and Levelt, 2004).

Previous fMRI studies investigating the categorically related condition either in comparison to the unrelated condition (de Zubicaray and McMahon, 2009; de Zubicaray et al., 2013) or to a control condition (de Zubicaray et al., 2001) have observed modulations in the BOLD signal in the left STG and MTG as a function of picture-word type. For example, a recent fMRI study (de Zubicaray et al., 2013) observed longer picture-naming RTs for related than unrelated stimuli, but a reduction in activity in the left MTG for related relative to unrelated stimuli, similar to our finding of reduced activity in the left STG for incongruent (i.e., categorically related) relative to neutral (i.e., unrelated) stimuli. In line with these findings, our results provide independent evidence of *increased* picture-naming RT and *decreased* activity in the left temporal cortex for categorically related picture-word pairs relative to unrelated pairs. This finding is also in line with a recent magnetoencephalography (MEG) study, which used very similar stimulus materials as in the present fMRI study (Piai et al., 2013). In the MEG study, responses from the left middle temporal cortex between 300 and 500 ms after picture-word presentation were *smaller* for categorically related (and congruent) picture-word pairs relative to unrelated pairs. Importantly, the behavioral data showed the usual pattern of *longer* picture-naming RTs for related than unrelated stimuli.

How can we interpret this difference between RTs and brain responses for related and unrelated conditions in the PWI task? In order to name a picture, speakers have to retrieve its name from long-term memory. Upon picture presentation, activation from the pictured concept spreads through the lexical-semantic network, leading to the activation of a cohort of words that belong to the network (e.g., Roelofs, 1992; Abdel Rahman and Melinger, 2009). Similarly, the distractor word also activates representations in this network. Crucially, in PWI, the picture activates the distractor word on related but not on unrelated trials. This “reverse priming” makes related distractors stronger competitors than unrelated ones (Roelofs, 1992). Such priming in the lexical-semantic memory system (e.g., Collins and Loftus, 1975; Roelofs, 1992) may explain why categorically (and semantically) related picture-word pairs show less brain activity in the left temporal cortex relative to unrelated pairs (de Zubicaray et al., 2013; Piai et al., 2013; and the present results).

Although this account can explain why we observed reduced activity in the left STG, it requires an additional mechanism to account for the slowdown in naming associated with categorically related picture-word pairs. Such a mechanism has been proposed by Roelofs (1992), who presented computational simulations demonstrating that the semantic interference effect in RTs is explained by reverse priming and selection of a word only if its activation exceeds that of alternative words by a critical amount. Moreover, the simulations by Roelofs et al. (2006) demonstrated that if the ACC is involved in enhancing the activation of a target concept until a corresponding word is selected, then the patterns of ACC activity in Stroop-like tasks (including those in the present study) can also be explained. Our fMRI results not only corroborate previous findings regarding the left temporal cortex, for which the activation reflects priming in the lexical-semantic memory system, but also highlight the involvement of the dorsal ACC, especially when selection and monitoring processes are more demanding due to the co-activation of categorically related words.

### Conclusions

The present study was designed to address whether a common neural-substrate might be engaged in the attentional control over linguistic and non-linguistic tasks with varying degrees of conflict. We observed activity in the dorsal ACC that was common to incongruent conditions of three different attentional control tasks, regardless of the response modality (vocal vs. manual) and nature of the stimuli (linguistic vs. non-linguistic). This common activation suggests a domain-general substrate that is called upon by all three tasks. More focused analysis of this commonly-activated region of the dorsal ACC in the linguistic-vocal tasks showed that it was sensitive to more difficult (i.e., incongruent) relative to easier linguistic stimuli. Finally, in the PWI task, increased activity was observed in the left anterior superior temporal cortex for picture-word pairs that did not belong to the same semantic category relative to picture-word pairs that did, probably reflecting the extent to which categorically related words were co-activated through target and distractor cues. These results suggest that language production engages brain areas implementing domain-general mechanisms for attentional control, as well as areas related to core language processes, such as lexical-semantic retrieval.

## Conflict of interest statement

The authors declare that the research was conducted in the absence of any commercial or financial relationships that could be construed as a potential conflict of interest.

## Appendix

**Table A1:** **Materials from the experiment (English translations between parentheses)**.

	**Picture name**	**Related distractor**	**Unrelated distractor**
Animals	Hert (deer)	Konijn	Piano
	Konijn (rabbit)	Hert	Drumstel
	Zwaan (swan)	Geit	Bus
	Geit (goat)	Zwaan	Fluit
Clothing	Jas (jacket)	Hemd	Molen
	Hemd (singlet)	Jas	Tafel
	Rok (skirt)	Trui	Neus
	Trui (sweater)	Rok	Banaan
Transportation	Auto (car)	Bus	Kanon
	Bus (bus)	Auto	Konijn
	Trein (train)	Fiets	Kast
	Fiets (bicycle)	Trein	Trui
Buildings	Kerk (church)	Fabriek	Zwaan
	Fabriek (factory)	Kerk	Zwaard
	Molen (mill)	Kasteel	Dolk
	Kasteel (castle)	Molen	Oor
Weapons	Dolk (dagger)	Zwaard	Arm
	Zwaard (sword)	Dolk	Been
	Kanon (cannon)	Pistool	Bed
	Pistool (gun)	Kanon	Kan
Kitchenware	Kan (pitcher)	Beker	Fabriek
	Beker (cup)	Kan	Pistool
	Bord (plate)	Glas	Rok
	Glas (glass)	Bord	Kerk
Furniture	Bed (bed)	Tafel	Gitaar
	Tafel (table)	Bed	Kasteel
	Bureau (desk)	Kast	Geit
	Kast (wardrobe)	Bureau	Molen
Body parts	Neus (nose)	Arm	Auto
	Arm (arm)	Neus	Peer
	Been (leg)	Oor	Appel
	Oor (ear)	Been	Beker
Fruit	Ananas (pineapple)	Banaan	Hert
	Appel (apple)	Peer	Fiets
	Banaan (banana)	Ananas	Trein
	Peer (pear)	Appel	Hemd
Music instruments	Drumstel (drums)	Gitaar	Bureau
	Gitaar (guitar)	Drumstel	Jas
	Fluit (flute)	Piano	Bord
	Piano (piano)	Fluit	Glas
